# Practices of Claiming Control and Independence in Couple Therapy With Narcissism

**DOI:** 10.3389/fpsyg.2020.596842

**Published:** 2021-01-25

**Authors:** Bernadetta Janusz, Jörg R. Bergmann, Feliks Matusiak, Anssi Peräkylä

**Affiliations:** ^1^Department of Family Therapy and Psychosomatics, Jagiellonian University, Medical College, Kraków, Poland; ^2^Faculty of Sociology, Bielefeld University, Bielefeld, Germany; ^3^Department of Child and Adolescent Psychiatry, Medical Colleague Kraków, Poland; ^4^Faculty of Social Sciences, University of Helsinki, Helsinki, Finland

**Keywords:** couple therapy, conversation analysis, narcissism, independence, vulnerability, sequence, topic, identity

## Abstract

Four couple therapy first consultations involving clients with diagnosed narcissistic problems were examined. A sociologically enriched and broadened concept of narcissistic disorder was worked out based on Goffman’s micro-sociology of the self. Conversation analytic methods were used to study in detail episodes in which clients resist to answer a therapist’s question, block or dominate the development of the conversation’s topic, or conspicuously display their interactional independence. These activities are interpreted as a pattern of controlling practices that were prompted by threats that the first couple therapy consultation imposes upon the clients’ self-image. The results were discussed in the light of contemporary psychiatric discussions of narcissism; the authors suggest that beyond its conceptualization as a personality disorder, narcissism should be understood as a pattern of interactional practices.

“Das erste steht uns frei, beim zweiten sind wir Knechte”

“The first is free to us, in the second we are servants”

Goethe

## Introduction

### Couple Therapy With Clients Who Have Narcissistic Problems

In this paper we investigate a set of interactional practices occurring in the context of initial couple therapy consultations with partners who have narcissistic problems. Because these patients have difficulties displaying weakness or need for help, they often deny the necessity of individual therapy and are more motivated to come to couple therapy due to the risk of losing their partner (Links and Stockwell, 2002). Furthermore, couple therapy with patients showing narcissistic conduct is of particular interest because long term relationships are regarded to have a stabilizing if not healing effect (Ronningstam et al., 1995; Lewis, 1998, 2000).

During couple consultations couples with narcissistic spouses often report basic communication problems and, accordingly, a significant level of stress. This is in line with the results of experimental studies that indicate that narcissistic spouses are highly problematic to their partners. They are described as showing hostility—e.g., criticism, insults—while discussing conflicts (Peterson and DeHart, 2014; Lamkin et al., 2017), as exhibiting aggressive behavior during competitive tasks (Keller et al., 2014), and as acting in an exploitative manner (Konrath et al., 2014).

Along this line, studies show that treatment of narcissistic personality disorder poses a huge challenge for couple therapy as well as for individual therapy. Yakeley (2018) reports rejection of diagnosis, feelings of unfair treatment or premature termination of therapy as serious difficulties impeding psychotherapy. A similar picture is drawn by Tanzilli et al. (2017) who identify the problem of establishing a good enough therapist-patient relationship as a main obstacle for individual therapy.

Couple therapy with a narcissistic spouse provides a specific naturalistic setting for a couple’s interpersonal spectacle (MacFarlane, 2004), which the therapist can witness as (implicit) addressee or overhearing listener (cf. Goffman, 1979). Lachkar (2004) highlights the circular, destructive patterns of communication in borderline-narcissistic couples that are enacted during couple therapy sessions. Links and Stockwell (2002) identify as a particular challenge the heightened defensiveness in individuals with narcissistic problems when a partner is witnessing an interpretation, or responding with disdain and anger for the therapist’s comments.

In diagnostic manuals, narcissism is conceptualized as personality disorder that characteristically includes impairments of self-functioning and predominant self direction, manifests in *“a pervasive pattern of grandiosity (in fantasy or behavior), need for admiration, and lack of empathy”* (APA, 2013). In clinical theories, grandiosity is understood as defense against an internal state of vulnerability (Kohut, 1971; Levy et al., 2007, 2011; Ronningstam, 2012). In contrast to this intrapersonal view, recent approaches conceptualize personality disorder as part of a dynamic system of interactions (Livesley, 2018) including interpersonal or situational factors. These factors can amplify individual personality predispositions, with the result that, for example, the presentation of a patient’s grandiosity may vary or oscillate during a therapeutic session depending on how secure the patient feels in the relationship with the therapist (e.g., Hopwood, 2018). Assuming the manifestation of narcissistic disorder, conceptualized as impairment in self-functioning, depends on certain social conditions, further research at the intersection between the internal world and the self in the social world is needed. To understand this, we need concepts that come from the sociology of the self.

### Self in Social Interaction

In our view, the clinical depiction of vulnerability in narcissistic personality, and the work of Goffman on the generic vulnerability of self in social interaction, ideally complement each other. As Peräkylä (2015) argued there is a yet unrecognized theoretical connection between Goffman’s theory of face and the psychiatric understanding of disturbances of self in personality disorders. This link between Goffman’s depiction of the self and contemporary clinical theories regarding narcissism implies that it is the experience of “face” that has been impaired in personality disorders, especially in pathological narcissism.

Throughout his writings, Goffman pointed out that whenever individuals engage in interaction, they necessarily display what they claim to be. In his early work (Goffman, 1955), he discussed this in terms of “face.” Face arises from the positive social attributes that a person, through her line of action in interaction, claims to herself, and that she expects others to ratify. In other words, Goffman contends, by anything we do in interaction, we claim a particular image of self either by saying or doing.

Goffman thus points out that the self is thoroughly social. For a person to be in good face, they need recognition from their interaction participants. Furthermore, the sociality of the self involves that we are not only sensitive to our own face and self, but also to the face of the other. The Goffmanian actor feels embarrassed also when it is the interaction participant who loses their face (Goffman, 1955).

In his 1955 essay and elsewhere, Goffman is very sensitive to the emotional meaning of the self thus claimed. Borrowing psychoanalytic terminology, he points out that we *cathect* our selves: we attach positive emotion to our self-image. But on the other hand, we are also inherently anxious about our self. The others may not ratify the self that we claim to be. This means that our face and our self-image is perpetually vulnerable (Goffman, 1983).

For Goffman, vulnerability of the self is an inherent by-product of social interaction: engaging in the interaction means accepting the risk of not being attended to, of not being ratified and responded to as what we claim to be (Goffman, 1955, 1971). The clinical theories of narcissism specify vulnerability of the self by pointing out that there are individuals who are, as it were, hyper-vulnerable. Since Freud’s essay “On narcissism” (Freud, 1957, orig. 1914) these individuals are called “narcissistic” insofar as they are utterly dependent on approval and attention by others (Kohut, 1971, p. 17) and in great need to be loved and admired (Kernberg, 1975, p. 227).

By investigating the interactions of narcissistic persons, we can see a “highlighted” version of the vulnerability that is there, in more implicit forms, in all social interactions. On the other hand, the Goffmanian way of understanding the omnipresence of self and its vulnerability in social interaction can help us to see more clearly self-related risks in our clinical materials.

### Analyzing the Self in Social Interaction: Conversation Analysis

In his publications, Goffman never dealt with psychotherapy, let alone psychotherapeutic interaction. Although his work on the intricacies of self-presentation in social interaction was enormously influential, he never based his studies on recordings of actual social episodes but relied mainly on ethnographic observations and occasionally on newspaper clippings or quotes from novels. This is where conversation analysis comes in.

Conversation analysis was developed as the microanalysis of the practices through which social order is generated by the interactants in the minutia of the unfolding social interaction in ordinary everyday life.^1^ In its early years, conversation analysis was focused on the identification and description of basic, if not universal mechanisms and devices of the organization of social interaction. It is a basic premise of conversation analysis that the various parts, which make up the interactional machinery, have the twin features of being context-free and context-sensitive (Sacks et al., 1974, p. 699). The principles of this interactional machinery regulate, e.g., the alternation of speakers, conversational repair, topic development, or reference to persons. They apply across different social contexts, but at the same time they provide opportunities for the participants to display their understanding of and orientation to the particular contextual conditions of the interaction.

The potential of conversation analysis for the study of interactants’ practices to particularize a social encounter prompted researchers to extend the area of study beyond informal everyday interaction and to include institutional talk, e.g., courtroom proceedings or emergency calls (Drew and Heritage, 1992), psychotherapy (Peräkylä, 2019), psychiatry (Bergmann, 1992, 2017), and of talk involving atypically developed participants, e.g., individuals with aphasia (Goodwin, 1995, Goodwin(ed.), 2003) or autism (Maynard, 2005). More recently, conversation analysis has been used as a method in studies on family and couple therapy, embracing interactional patterns in the establishment (Sutherland and Strong, 2011) and ruptures and repairs (Muntigl and Horvath, 2016) of the therapeutic alliance, in circular questions (Diorinou and Tseliou, 2014), and in interactional asymmetries (Janusz et al., 2021).

Conversation analytical research has also picked up Goffman’s idea of self in social interaction. Although his concept of self does not directly translate into detailed conversation analytical observations, conversation analysts have started to investigate specific contexts of action and sequential environments in which the situated identities of participants become relevant or participants orient themselves to face problems (see Maynard and Zimmerman, 1984; Lerner, 1996). Studies thus far have demonstrated that participants’ orientation to issues of face concerns only specific moments of interaction. In their analysis of agreements on assessment sequences, Heritage and Raymond (2005) suggested that claims and sometimes disputes regarding knowledgeability involve not only epistemic issues such as social expectations, rights, and obligations to know but also issues of face. However, Goffman’s radical claim regarding the *omni relevance* of face has not been met with empirical evidence from CA studies, which focus on clearly definable conversational objects.

According to its epistemological stance, conversation analysis abstains from judgments about the facticity of impairments of self-functioning, of narcissistic personality disorder, or other clinical conditions; it cannot contribute directly to our understanding of personality disorder. It can, however, analyze when and how a participant’s behavior becomes “noticeable” for the co-participants—and for the researcher—as unexpected, inappropriate, improper, and, thus, as possibly non-normal. Based on this procedural conception we do not ask “what is and who has a narcissistic personality disorder,” but instead we ask “when” is a narcissistic personality disorder.^2^ Thus, our main focus is on when, where, and how an activity occurs in a couple therapy session that clinicians will identify as features of narcissism.

The focus of our paper is on the question how clients who have narcissistic problems act in the interactional context of couple therapy. Particularly we seek to analyze in detail the activities of these clients in situations in which they are expected to answer personal questions. And we closely look at how they respond when their personality or behavior is commented upon by the other spouse or the therapist.

## The Method

### Participants

The data set, with which our study started, comprises the initial therapy sessions of seven couples who all reported problems in their marital relationship.^3^ For each of these couples at least one spouse was diagnosed as showing features of personality disorders. In four of these couples, one spouse respectively (three men, one woman) was diagnosed with narcissistic features. These four couples together were taken as database for this paper.

All the therapists had systemic training in communication, structural systemic theory, and the Milan approaches, which was their primary therapeutic approach. Yet they also had additional training in psychoanalytic or psychodynamic therapy.

### Research Setting

The decision to include only first consultations in the dataset is based on the fact that, within the systemic framework, first sessions are usually more structured than the following therapeutic sessions, which facilitates the comparisons between cases. In systemic couple therapy first consultations, therapists encourage the spouses to define their therapy goals and desired changes, actively investigating how the complaints may be influenced by the couple’s interaction. In the Milan approach the circular questions are used while gathering the information about the relational patterns in the family; the therapist’s aim is to observe what may prompt change in the interactions (Selvini et al., 1980). In structural approaches the therapist is expected to observe the family transactions, particularly those related to the presented problem, as well as organizing the interview in such a way that the therapist’s leadership is the source of safety and confidence for the couple (Weber et al., 1985; Nichols and Tafuri, 2013).

The issue of safety is particularly important during the first sessions. The spouses are faced with the difficulty that they have to talk to a stranger—the therapist—about their most private marital affairs, their disappointments with each other, their common history of conflicts, their mutual injuries and transgressions, their most intimate wishes, hopes, and experiences. Usually both spouses are aware—or at least sense—that each of them has a share in the turmoil and jeopardy of their marriage. And even though the therapist’s role is to reformulate the “blaming utterances” in terms of problematic relational patterns, interactional studies show that this kind of circular perspective may contribute further to developing blaming conversational sequences (Patrika and Tseliou, 2016).

Taken together, the constellation of a couple therapy implies for both clients that they have to cede control of the image they want to preserve. And this situation is considered as particularly threatening for clients who already have difficulties in receiving and maintaining recognition of their ideal self image (Links and Stockwell, 2002; MacFarlane, 2004).

### Method of Analysis

In analyzing early exchanges between therapist and clients, we were guided by the methodological principles of conversation analysis, i.e., at the first stage of analysis, the data were studied in an “unmotivated way” (Sacks, 1992, p. 175). The fact that the examined conversations took place during a psychotherapeutic session played initially no role in the analysis of the data, and the clients’ utterances were not scanned for clinical symptoms. The researchers made any effort to avoid speculating about the clients’ intentions or goals, instead they committed themselves to stick to the maxim of observability. The validity of a description had to be attained by referring to an observable detail in the ongoing interaction. In this, the researchers followed the ethnomethodological “study policy”, to treat everyday activities as members’ methods for making those same activities reflexively “accountable,” i.e., observable and describable (Garfinkel, 1967).

Based on video recordings of the psychotherapeutic sessions, major parts of the core data set, and additional consultations, were transcribed according to the established transcription system in conversation analysis, originally developed by Jefferson (1984). The analysis started by “making an observation”. What struck the researchers’ attention were moments in which the interaction ran off in an unexpected way: Something unusual happened, a manifest interactional “hitch” occurred, an interruption of the flow of interaction, a client’s noticeable intervention, or some other infraction of a conversational rule. Particular attention was given to the ways in which the participants created these conspicuous moments or contributed and responded to them, and projected—as explicit statements or questions, as presuppositions, or by implication through their actions—positive or negative attributions regarding the self of themselves and each other.

A collection of these noticeable events and their interactional management was made, still without any clinical interpretation but with an eye to the question, how these events are related to the interactional positioning and self images of the participants. Various episodes were analyzed turn by turn with regard to their interactional unfolding and with the aim of identifying and disentangling various meaning layers of an utterance in a given sequential environment. Eventually, “interactional control” was identified as the common thread, running through the collected episodes. The concept of interactional control pertains to activities of clients with which they resist interactional dependence on others (therapist or spouse) or stipulate the conversation’s further course. The analysis showed that controlling activities are prompted by threats to the clients’ interactional self-images, and serve as means to manage such threats and to maintain the purported self.

### Procedure

The data of our study was gathered from couple therapy first consultations that were conducted in the Family Therapy and Psychosomatics Department, Medical College, Jagiellonian University Krakow.^4^ In the department, therapy sessions are regularly video recorded for the purpose of training and supervision. The cases that make up our database were selected by the therapists who identified couples that were particularly difficult to talk to. As a result, in the course of the therapy the therapist came to the conclusion that a personality pathology might be lingering in the background. Couples who were identified by the therapists as meeting the criteria were informed about the research project and were asked about their willingness to participate in it. Those who agreed to participate finally signed the statement of agreement. The narcissistic symptomatology was on the level of Personality Disorder. The initial diagnosis of Personality Disorder was later on confirmed by formal testing with the Shedler–Westen Assessment Procedure SWAP (Shedler and Westen, 2007).

In the next step the transcribed video recordings of four first therapeutic consultations were analyzed. As a result of the data analysis the phenomenon of interactional control in NPD couples emerged as the main object of our study. In order to find out whether these phenomena occurred more than just once, we went again through the recordings of the four sessions that were the data of this study. For comparison, we also dealt with in an unsystematic manner other recorded sessions with spouses that were not diagnosed with narcissistic personality disorder, but since this was done in an explorative mode it was not included in this paper. The distinct controlling practices in narcissistic spouses were not identified in spouses indicating other than narcissistic personality problems, and in spouses without PD related problems. However, a systematic comparison needs to be done in future studies.

## Results

The controlling practices employed by the spouses with narcissistic problems pertain to the sequential position in interaction, to the management of the topical flow of the conversation, and to the display and enactment of identity. In the following, these three areas of practices will be dealt with one by one, although actually they overlap and are intertwined, which is why the same examples are sometimes used for different analytic purposes.

### Controlling the Sequential Position

In our data, the clients repeatedly make moves whereby they interactionally sidestep from being in the responsive position. Generally in social interaction, every sequence-initiating utterance stipulates the type and range of subsequent activities. By asking a question, a speaker generates an expectation for the recipient to answer and restricts the terms of his/her response. Thus, a recipient is strongly constrained by the question and its formulation, its mode and its presuppositions; the recipient’s turn is “sequentially dependent upon the previous one” (Schegloff, 1968, p. 1076).

In psychotherapy, the question–answer–regimen is loosened; questions are not formulated as pressing requests for information but as invitations to cooperate by volunteering an answer. Indeed, in the following segment, (The meaning of the transcription symbols in the Appendix).


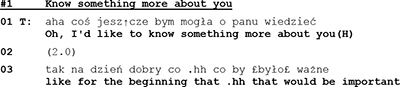


-the psychotherapist does not even formulate a question but states her wish to know more about her recipient, the husband. And since the husband remains silent for 2 s, she continues by telling him why his participation is important.

The psychotherapist’s unobtrusive move to coax husband to talk about himself is only partly successful. Client does respond, but he does not answer the therapist’s question.


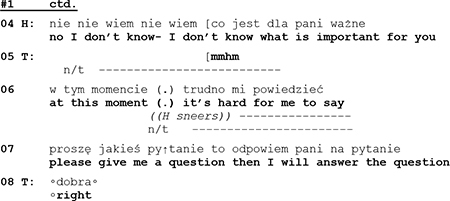


In his rejection [“No”] the client takes issue with two features of the therapist’s initiative move. He refuses the opportunity to decide by himself what is important for him and what he would like to talk about. And although the client, by formulating a counter request, does respond to the therapist’s question, he does not answer it. He resists the conditions that are set and controlled by the therapist’s question, and formulates for his part the conditions under which he would be willing to answer. Thus, his sidestepping response and counter request can be seen as a move to control the terms of his participation.

It is not unusual in everyday interaction that recipients, instead of answering a question and thereby implicitly accepting its legitimacy, try to resist the constraints of the question-answer format and alter the course of interaction (Stivers and Hayashi, 2010; Heritage and Raymond, 2012). In many people-processing organizations, interaction consists of a series of question–answer-sequences (Drew and Heritage, 1992), and although clients are expected to stick to the conditions of the question, they often sidestep or resist questions as has been shown for police interrogations (Jol and Stommel, 2016), news interviews (Carranza, 2016), counsellings (Muntigl, 2013), psychotherapy (Yao and Ma, 2017), and other institutional contexts (Chevalier and Moore, 2015).

However, H’s reluctance in extract #1 to submit to the sequential ties of a previous question is not a singular, but a recurring event that can be observed as a habitual pattern in many other instances in this therapeutic session, as in the following segment:


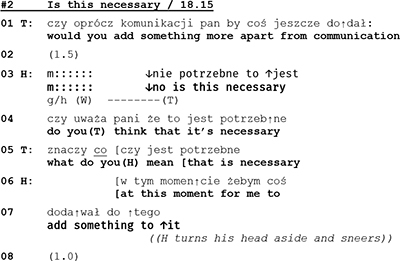


About 15 min into the first session with a couple at the age of about 40 years, the therapist invites the clients to tell more about their reasons to come to therapy. After the wife has provided some information about herself and her view of the couple’s problems, the therapist turns to the husband and asks for his supplementary statement. Instead of answering by giving the information asked for, the husband responds with a counter question requesting to know whether this is necessary. Research has shown that there is a dispreference for patient-initiated questions in physician-patient encounters and that questions that are nevertheless asked by patients are modified in order to indicate their dispreferred status (Frankel, 1990). In segment #2 the client’s counter question is not marked as a dispreferred activity. With his inquiry about the necessity of the therapist’s question, he not only challenges the therapist’s professional authority but steps out of the interactional space in which his action is controlled by the therapist’s preceding question. By asking a question himself, he occupies a sequential first position, thus making an answer by the therapist “conditionally relevant” (Schegloff, 1968, p. 1084) and exerting for his part control of the therapist’s subsequent action.

In one of his early lectures (1964) Sacks remarked that “the attempt to move into the position of ‘questioner’ seems to be quite a thing that persons try to do. (…) As long as one is in the position of doing the questions, then in part they have control of the conversation” (1992, p. 54). And with regard to adult–child interaction Mishler(1975, p. 99) has observed that “when adults initiate a conversation with a question, they retain control over its course by successive questioning, (…) when children ask an adult a question, the adult regains control by responding with a question.” It seems obvious that the question–answer-sequence has an inbuilt logic of control. Questions not only stipulate that a response is due but also determine what kind of answer is expected.^5^ As we have shown, it is a characteristic of narcissistic clients that they “break out” after a personal question by side stepping responses or counter questions. With their maneuvers of resistance they mark the psychotherapist’s preceding question as an infringement of their autonomy, and, at the same time, conspicuously re-claim their independence.

### Controlling the Topic

In our data, clients control and restrain the topical flow of the therapeutic conversation. Below, we will show practices whereby this is accomplished.

Participants in a verbal interaction always talk “about something,” and what they talk about constitutes the “topic” of the conversation. In general, topic is characterized by two complementary components that together form a contradictory unit (Bergmann, 1990). On the one hand there is a constraint that ensures that there is a topical flow at all. This constraint of progressivity imposes on every speaker the obligation to contribute something new to the ongoing verbal exchange. On the other hand the obligation to introduce new items is counterbalanced by the constraint not to chuck in just any new matter but to stay on topic and to show consideration for the maintenance of the conversation’s actual topic. Topic development usually is the outcome of the co-interactants’ cooperation, but a participant may use stricter “topic control to avoid the gainsaying of troublesome evaluations” (McKinlay and McVittie, 2006). The more detailed organization of topic is dependent on the type and institutional purpose of the encounter.

In couple therapy sessions, one way for the clients to contain and dominate the conversation’s topic is by persevering and insisting on one’s own point of view, an example of which can be found in the following segment.


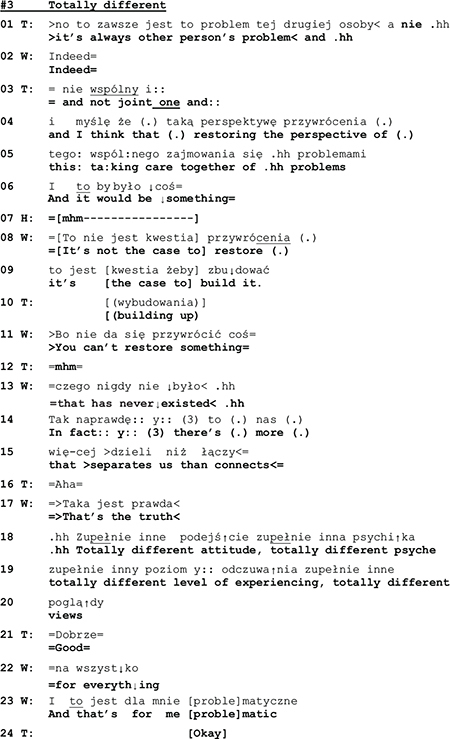


Through a repair practice that comes close to “lexical substitution” (Rae, 2008) the wife rejects what the therapist’s said about of restoring the common ground of the couple’s life *(l.8 “It’s not the case to restore”)*, and introduces an alternative version of the marital state of affairs (*l.9 “it’s the case to build it”).* In her subsequent utterances (l.11, 13, 14–15) she emphasizes and explains her view before entering into a monolog with a list of differences between her and her husband. This list is instructive in two ways. On the one hand the list is built as a series of extreme case formulations (“totally …”) that are used “in anticipation of non-sympathetic hearings” (Pomerantz, 1986, p. 227), to underline the rightness of a case and to forestall possible refutations. On the other hand W’s list is remarkable insofar as it is constructed out of four items (l.18–29) and as such it deviates from the “three-partedness” that Jefferson(1990, p. 89f.) has shown to be “a basic structural principle” of lists. With the twofold overdoing of her case, W. clearly marks that for her this issue is non-negotiable and not worth talking about any longer; for her the topic is closed.

The following two extracts show yet another type of topic control. In these instances, topic control occurs after a problematic issue was brought up and described by the spouse. At the beginning of Extract 4 (l.1–3) the wife describes the husband’s state of mind that she views as problematic.


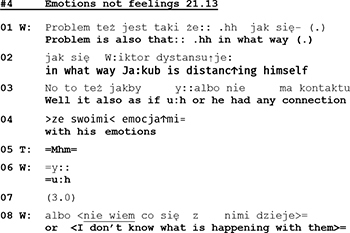


Although his wife is talking about him, the husband does not take the opportunity to respond but remains silent (see pause of 3 s in line 07). Only when his wife points out her inability to understand her spouse’s mental and emotional life *(“I don’t know what is happening with them”)*, he offers a comment:


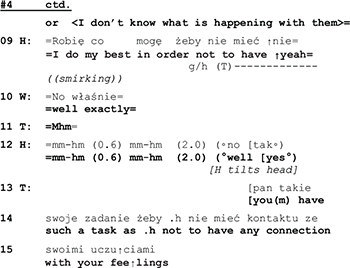


With his statement *“I do my best in order not to have them”* the husband transforms that which his wife has just described as a problem into his achievement. But with his smirking face he frames his utterance as a funny remark, and he even looks at the therapist, thereby apparently monitoring her response and possible appreciation. In case of success, a funny remark generates joint laughter, which in turn regularly leads to a termination of the topic at hand.^6^ However, in the extract above, instead of laughter, his wife reacts with a comment that displays “knowing” (l.10: *“well exactly”*), and the therapist, in her response, treats his utterance as a serious statement, ignoring its ironic sub-meaning. In the end, his joke did not terminate the subject. The misalignment between the therapist and the husband’s actions continues over the next turns:


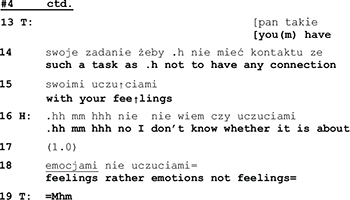


The husband starts answering hesitantly (l.16), he expresses doubt as to the appropriateness of the therapist’s wording and, after a pause of 1 s., continues by correcting the therapist’s choice of words (feeling vs. emotion). While the semantic significance of the repair remains unclear, it is interactionally consequential in two ways: it induces an interruption of the topical flow (Egbert, 1997), thus releasing the husband from having to respond to the issue brought up by his wife (connection with his emotions). And by rejecting her vocabulary the husband furthermore calls into doubt the therapist’s professional competence and displays unwillingness to enter into a therapist-patient relationship with her.

An even more powerful and bold practice to take control of the conversation’s topic can be found in the following extracts. Above, we examined these extracts regarding the control of sequence; yet the same examples also involve control of topic. Despite the fact that it is the therapist’s task to lead the conversation and guide the couple through this first session, we observed in our data several instances in which the patient acts in such a way to decide the subject of the talk and how it should be approached. In the following example, the therapist’s request for basic personal data from the husband leads to a silence of 2 s.


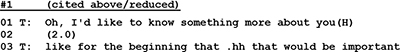


The silence is terminated by the therapist who continues by expanding her question and by underlining the importance of the husband’s participation. In his subsequent response, the husband refuses to give an answer by pointing to his lack of knowledge regarding the therapist’s expectation (l.04). Directly after that, the husband instead asks for a clear cut question from the therapist in order to deliver the requested information (l.07):


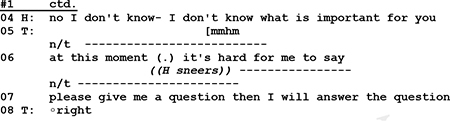


Whereas extract (02) shows the husband’s resistance to enter into topical talk according to the therapist’s stipulation, the following extract (01) captures an episode in which the same patient blocks the therapist’s initiating move by redirecting the topical focus away from him to the therapist.


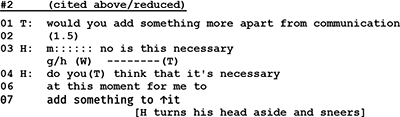


After a pause of 1.5 s and a hesitation marker (*m::::::*) the husband first rejects the therapist’s question and continues to sidestep an answer with a counter question inquiring after the topic’s necessity. Instead of talking about his perspectives and problems, he initiates a move to transform the conversation into a meta-discussion about the necessity of the therapist’s question—contesting, thus, her professional authority.

To summarize: How and in which direction the topic of a conversation develops in the flow of talk is in many ways unpredictable for the co-interactants. In the situation of a couple therapy clients may find themselves in awkward situations because the subjects that were brought up jeopardize their ideal self and invoke their vulnerability. As we have shown, clients apply various methods to gain control of the conversational topic, with the effect of diverting or forestalling talk about issues that could threaten their face. They can insist on a subject by extended and monologic utterances, or they can block the further development of the topic by eliciting laughter with a funny remark or a joke. The most blatant mode of steering the conversation away from a threatening topic is to engage the therapist in meta-talk by casting doubt on the therapist’s entitlement to know and to ask questions about personal issues.

### Controlling the Displays of Identity

According to Sacks et al. (1974), a key aspect of the turn taking machinery of conversation is that it can accommodate “interaction between parties with any potential identities” (p. 700). Social identities of participants of conversation are brought into being through their ways of operating the turn-taking system. Sacks et al.(1974, p. 718) highlight the local transformations of such identities: the machinery of conversation “is compatible with multiplicities of, and changes in, the social identities of some ‘same’ participants.” In what follows, we will examine such multiplicities of clients’ identities in couple therapy.

A distinction is often made between two facets of self and identity. One facet has to do with what is explicitly said or believed about a person, and the other facet has to do with what a person experiences or conveys about themself through their actions—without necessarily putting into words these things (see Goffman, 1955; Neisser, 1988; Leary and Tangney, 2012). Bamberg (2007); Deppermann (2015), and Deppermann et al. (2020) broadly distinguish between “told self” and “performed self”—a distinction that we find particularly useful in the study of couple therapy and that we will adopt in the following. “Told self” involves the verbalized attributions that the spouses make about themselves and each other; “performed self” involves what they convey about themselves through their actions.^7^

In first sessions of couple therapy—like those that we use as data—issues of identity are particularly pertinent. The therapist’s primary task is to learn to know the couple: who the spouses are and what is their problem. For the therapist, the told self—what the spouses tell about themselves—is important, but at least equally important is the performed self, i.e., what the spouses convey about “who they are” through their actions.

We will now go through our extracts once more, re-elucidating them from the point of view of identity construction. Let us consider once again Extract 2 shown above. The therapist requested the husband to tell her more about himself, and the husband declined to answer:


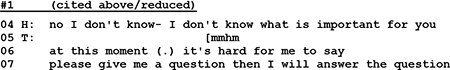


The husband does in effect refuse to tell about himself: thereby he withholds any further specification of his told self. In terms of the performed self, however, the husband is much more active. Refusing to answer the question is a powerful move in performative self-presentation: the husband displays that he is not someone that is controlled by the therapist; he highlights his independence from the therapist. In this context it also can mean that he is not one who would be seeking help. Thereby, he claims and demonstrates strong independence.

Extract 2, also shown above, involves identity construction that is very similar to that in Extract 1. Again, the husband declines to disclose more about his problems or the problems of the couple as he sees them, and thereby, he withholds further specification of his told self.


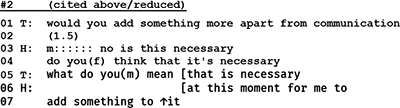


In terms of performed self, his counter question (lines 3–4) shows, like in Extract 2, that he is not controlled by the therapist. The specific context where the husband now claims independence is of importance: in line 01, the therapist is eliciting description of the couple’s problems, as seen by the husband. By the very act of declining to answer, the husband conveys something about his relation to problems: as he has neither the need nor the will to specify problems, he also shows that he has not burning problems, at least such that could be dealt with here, in couple therapy.

In the cases shown above, the most intensive identity construction seems to take place in the performative rather than declarative field. Consider now extract 5 shown below, where the issues of told self are central. In the closer look, however, performative aspects of identity are equally important also here. Shortly before the exchange that is shown in Extract 4, the wife has complained about the husband’s habit of smoking marijuana (data not shown). In Extract 5, the husband challenges this.


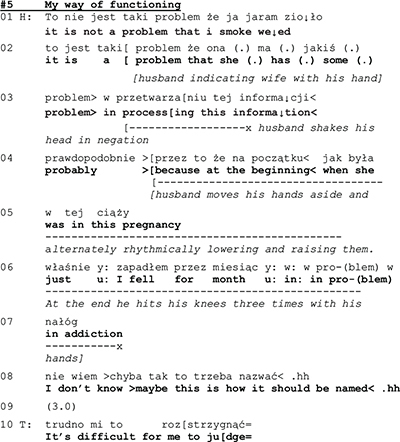


The husband engages in a complex description of the couple’s problem, whereby he also conveys a self-description. First, in line 01, he rejects the wife’s problem attribution (data not shown), and then in lines 2–3 claims that the problem is in fact in the wife’s inability to understand the smoking. While the wife’s prior complaint ascribed an identity of “problem source” to the husband, he now makes a counter-ascription, claiming that the wife is the problem source. The husband continues his account by admitting that he has had an addiction. The admission is couched by minimizing devices, such as temporal reference (for a month) and relativizing the categorization “addiction” (*maybe this is how it should be named*). On the level of the told self, the husband thus builds an image of himself of a non-problematic marijuana user, mistreated and misunderstood by his wife—thus, as a victim rather than the evil-doer.

A few seconds later, the husband again makes moves that entail an identity of a victim. In response to what the therapist said at the end of the previous extract (line 4) about the difficulty for him to judge (whether husband’s marihuana use was an addiction), both spouses assert that they don’t expect the therapist to judge (data not shown). This prompts the therapist to ask what they think the therapy is about (line 16 below). The wife’s answer is that they would learn to communicate (lines 16–17).


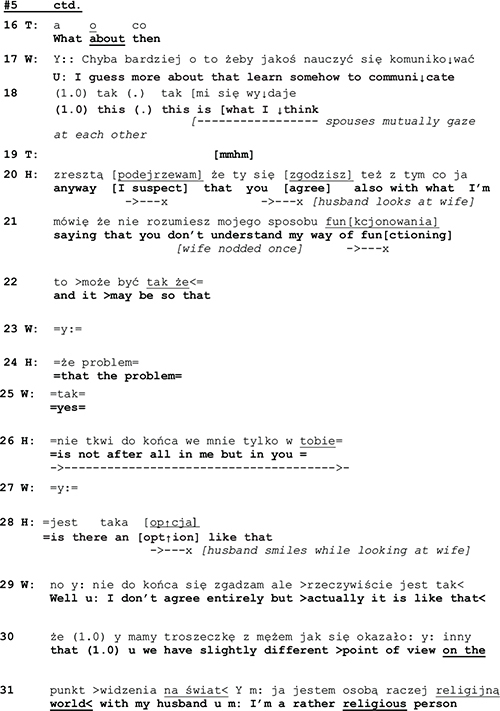


Rather than taking stance to W’s suggestion, or in some other way dealing with the therapist’s question, H returns to his earlier arguments, pointing out that W does not understand him (line 20) and that the problem is in W rather than in H (22–23, 25). Again, he assumes the identity of a victim.

As the told self that the husband claimed as being someone who was misunderstood by his spouse, not the source of the problems, but rather a victim of his wife’s behavior, the performative identity looks rather different. The husband is engaged in a self-defense that he delivers in an agitated way, speaking quickly, in high pitch, and gesticulating brisky while he talks. He delivers his utterances in a self-initiatory way, and not as answers or other responses to the therapist’s initiations. This sequential and topical control is most clearly to be seen in the latter part of extract 4, where the husband fails to respond to the therapist’s question (l. 16) and W’s answer to it (l. 17–18). Rather, he pursues his own self-initiatory agenda. And even more: he designs his (re-)definition of the couple’s problem in lines 19–20, 22–23, 25 as a claim for the wife to agree with, thereby departing from the normative turn-taking system of couple therapy (where the therapist is the one who asks questions). By this self-initiatory action where he takes the first position in the sequential organization, the husband displays an identity. In spite of the declarative claim of being a victim, his performance constitutes him as an independent actor.

Let us consider now another example where the told self is on the surface of the interaction, and yet identity work is equally done in terms of the performed self. We will return to extract 3 discussed above. At the beginning of the extract, the therapist is describing a “typical” way of experiencing problems in couples—seeing the problem in the other spouse—and then depicting the apparently better alternative in the “perspective of restoring” (l. 04–05) and joint care of the problems (line 05).


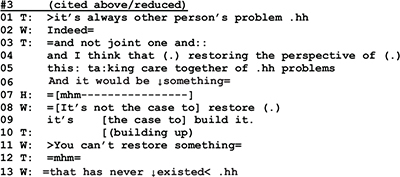


While the husband in line 07 seems to at least minimally agree with the therapist’s formulation of the alternative perspective, the wife in lines 08–09, 11, and 12 refutes it. The refutation has first a kind of positive edge in it, as she in line 09 talks about building up (something new) as an alternative to “restoring.” However, as she continues her utterance (lines 11 and 12), she shifts the referential focus and emphasizes the negative, that there is nothing to restore. Thereby, she starts to build a told self for herself and the husband as an inherently unhappy couple. This building of the negative identity is intensified as the wife continues her talk:


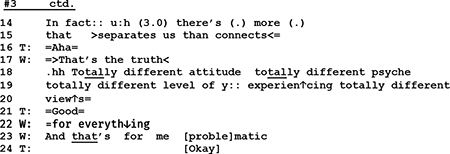


As we have shown above, with a list of descriptions employing extreme case formulations the wife in lines 14–24 depicts the couple as a lost case. Now if we turn to the performed self, a rather different picture emerges. As it was argued in the prior section, W takes in extract 5 the control of topic and control of sequence to herself. By changing the topical perspective (from expectation of restoration to lamentation of failure) and by moving from a responsive position prepared for her by the therapist, to the first position through her emotionally loaded self-disclosure, she displays interactional independence from the therapist. This independence is colored with what might be termed “passionate honesty,” as assumes the openly negative attributions for the couple.

In the beginning of this section, we made a distinction between told and performed self. The cases examined in this section showed that the *told* selfs claimed by the participants were rather variable: In extracts #1 and #2, the patient avoided identity avowal; in extract #5, the patient presented himself as a victim and located the problem in the spouse; whereas in extract #4, the patient actively assumed problems as part of the couple identity. While the picture of the told selfs was thus variable, the examination of performative selfs showed a more unified picture. In all cases shown above, the patients’ performative selfs foregrounded their independence of the interaction at hand, and hence, of their interaction participants.

We suggest that this performative claim to independence is as important, if not more important, than the variable declarative claims, in the clients’ identity work and self presentation in the first consultations. Furthermore, we suggest that the claim to interactional independence is strongly context dependent. Couple therapy consultation as a social situation involves a possibility of dependence: the couple is there to seek help. The local sequential contexts that we examined above involved more specific possibilities for dependence, especially when the spouse attributes problems to the client. Therefore, we suggest that the claims to independence are prompted by *risks of dependence*, emerging in the couple therapy interaction.

## Discussion

In this paper, we have described, using CA, three facets of interaction—sequence, topic, and identity construction—where narcissistic clients in our sample can be seen to exert interactional control. We observed,

(1) that in situations in which clients find themselves obliged to answer a personal question, they often resist and, by stepping out of the dependent sequential position, take control of the interaction engine;
(2) that in situations in which the consultation is about to turn up unfavorable and threatening subjects, clients often make steps to control and stipulate the direction of the therapeutic talk; and
(3) that in situations in which clients face the danger that their self-images may become precarious and form cracks, they take measures to maintain in their expressions and actions a presentation of themselves that foregrounds their independence.

The clients’ controlling practices pose different challenges and difficulties for the couple therapists. There seem to be two key areas of the therapeutic work that these difficulties pertain to: the ability of disclosing weaknesses and personal problems and related to it establishing the therapeutic relationship.

### The Problem of Establishing the Therapeutic Relationship

Bordin’s classical concept of therapeutic alliance (Bordin, 1994) involves engagement in collaborative, aim oriented work, as well as developing reciprocal, interactive relationships. We suggest that the clients’ display of independence can involve a major challenge for the development of the “micro-level” therapeutic collaboration.

One aspect of Brodin’s theory concerns the working alliance. It means that building up an alliance is an active, sometimes implicit process of negotiation that starts from the very beginning of treatment and is renegotiated in the course of the subsequent therapeutic sessions (Bordin, 1994; Hatcher and Barends, 2006). The practices of clients with narcissistic problems by which they deny interactional dependence and control the course of the encounter may block this “implicit process of negotiation.” These practices very often induce—in the therapist as well as in the spouse—silence, hesitation markers, verbal disfluency, and other displays of momentary confusion, that can indicate micro-level difficulties with building up the “reciprocal, interactive collaboration” between all participants of couple therapy talk.

The other aspect of alliance, according to Bordin’s theory, pertains to the affective bond. The clients’ controlling practices may also be seen as blocking the evolvement of the affective bond between them and the therapist that is constituted by mutual dependence, and that contribute to the collaborative work. Through their controlling practices, the clients mark their own independence and authority, but at the same time they implicitly display their disregard for the therapist’s face by correcting his/her vocabulary or by undermining his/her agenda.

Focusing on the development of the therapeutic alliance at the initial stages of treatment, Ronningstam (2012) suggests that narcissistic patients are prone to provoke and control the therapist, while Dimaggio et al. (2006) depict their tendency to power struggle. Our observations are in line with this, but our study furthermore shows that the very interactional organization of the first counseling session with its overall question–answer structure arrests the clients within a frame of (sequential) dependence that may add to their resistance and obstruction of the development of the alliance.

### The Problem of Addressing and Disclosing Weakness

Resistance to disclosure of one’s personal affairs tends to happen early in psychotherapy, mostly already during the first couple consultations. In disclosing the couple’s problems, the spouses often locate the problem in the other spouse. They also take defensive positions while being described by their partner as the source of marital problems. Our study has shown that clients with narcissistic problems seem to use more specific strategies to thwart the disclosure of their personal problems. In our view, these practices can be traced back to the client’s anticipation that uncontrolled topic talk, with its soft and inconspicuous transition from one subject to the next, may disclose weaknesses or flaws and is therefore perceived by clients as “risky.”^8^

Clinical literature suggests that narcissistic individuals are particularly sensitive to threats to self-esteem (Freud, 1957). Higher rate of psychotherapy dropout among narcissistic patients has been understood as reflecting efforts to manage self-esteem (Ellison et al., 2013). It is in line with classical observations of Abraham(1927, orig. 1919) who described the tendency of narcissistic patients to actively disrupt interventions that threaten their grandiose self-image.

The risks that a client may anticipate concern his/her self image and can arise from three intertwined contingencies. To begin with, the therapist, based on his/her institutional authority, is entitled to define the conditions of talking and to ask personal questions—an asymmetry that clients with narcissistic problems can perceive as a threat of their independence. Second, the spouse can be an additional source of threat for the client’s face as he/she is witnessing how he/she talks about their marital situation; he/she is also a witness of the therapist’s comments and may furthermore build a temporary coalition with the therapist (Janusz et al., 2021). Third, as we have shown, a client may perceive the unrestrained topical flow of the therapeutic talk as threatening since it could lead to statements or stories revealing about his/her problems or weaknesses.

The co-occurrence of the risks to self and the controlling activities in our data may be interpreted in light of the classical clinical debate regarding vulnerability and grandiosity in narcissism. Grandiosity manifests itself in therapeutic sessions seldomly as “grand grandiosity,” i.e., as boasting and bragging, but more often as display of momentary superiority and interactional dominance. Exercising control is one of the forms in which “small grandiosity” may appear; the one who is in control can bask in his/her supposed admirability. Kohut (1971); Kernberg (1975), and Levy et al. (2007, 2011) suggested that in narcissistic patients, the grandiose mental states oscillate or even co-occur with vulnerable mental states. On the basis of empirical studies, other authors (Cain et al., 2008; Pincus and Lukowitzsky, 2010) have pointed out that there are particular social contexts that intensify the duality between grandiosity and vulnerability, resulting in self-esteem dysregulation. First session in couple therapy might be one such context.

The dialectics of risks to the self and controlling activities are not something that we would expect to find only in the environment of couple therapy and with narcissistic persons. Goffman (1955) suggested that all interactions bring about risks to the participants’ selves, and that such risks are normally responded to through corrective work—face work—to restore the threatened selves. Couple therapy with narcissistic patients can be taken, therefore, as a “prism” that makes particularly salient and noticeable dynamics of self in social interaction, that are there in all social encounters.

### Limitations of the Study and Suggestions for Future Research

It can be argued that couple therapy first consultations are awkward if not threatening to all patients, not only narcissistic ones. Clinical experience suggests that the first consultation is particularly menacing for narcissistic patients. Yet, further systematic studies comparing narcissistic and non-narcissistic patients are necessary. An additional study would be most interesting in which first and later consultations are compared with regard to the question whether clients with narcissistic problems continue with their controlling practices or learn to let go and loosen their defensive habit.

A further line of research that we were unable to pursue arises directly from the constellation of couple therapy. Though we identified the controlling practices of narcissistic clients as interactional maneuvers, we did not take the triadic structure of the couple therapy interaction systematically into account. In most cases, our focus was on the interaction between the therapist and one spouse, rather than on the triad. But the simultaneous presence of therapist and intimate partner in which always one of them is the addressee whereas the other is the passive listener, is certainly relevant with regard to the practices of presenting, and sustaining to display, a coherent ideal self image. Research on the ways in which controlling practices affect triadic therapy constellations and are affected by it would be a necessary and most intriguing complement of our study.

A critical issue lingering through our entire text pertains to the question how statements about the interactional realm (controlling behaviors, interactional risks) can be linked to statements about the internal realm (personality related dispositions, perceptions of the risks for the self, narcissism). This is a big and not least philosophical issue and can, of course, not be tackled in a single—and moreover empirical—study. In our study we have proceeded under the assumption, which is a principal methodological presupposition in CA, that no one can look behind the forehead of another person and therefore the other’s mind is for no one directly accessible.^9^ On the other hand, in the everyday world we are able to “see” the intentions of others, to “read” their minds, since we have no other choice than to equip their behavior with meaning^10^. In that sense, the mind of others is “transparent” for us (Coulter, 1977). Ethnomethodological and conversation analytic studies deal with “cognitive” issues such as expectations, memory, or perception, but these issues are always and only dealt with as observable activities. Along this line we approached “narcissism” as an object that is accomplished and realized in and through interaction. This in accordance with more recent psychological theories (Hopwood, 2018; Livesley, 2018) in which it is argued that personality disorders cannot be conceptualized as an intrapersonal feature only, but must also be seen as a phenomenon in the interpersonal context. Yet, we also acknowledge that mind, as a subjective experience that emerges in interactional contexts, is real and relevant for the understanding of personality disorders. Based on our co-constructive view on personality disorders, two questions arise that might be topics for future studies.

The diagnosis of “narcissism” (or any other personality disorder) is the final outcome of a series of tests, interviews, and other assessment procedures through which professionals are able to identify and “read” observable interactional events as “signs” or “evidence” of an unobservable intrapersonal condition, i.e., as a symptom of a hidden pathological disposition (Bergmann, 2017). On the other hand, laypersons observe each other in all interactions, also making some kind of colloquial personality assessment. Little is known about the question how the mode of professional diagnosing and the mode of lay assessing are related to each other. A study is needed in which the divergent logics of psychological assessment in lay and professional contexts are laid out and reconstructed.

A second, parallel study would be necessary in order to shed light on the respective epistemic status of the peculiarity/impairment that occasioned the demand for psychotherapeutic help. Usually, the professional program of testing and probing leads to a diagnostic category in which the contributions of the test procedures and of the social interaction with the test personnel has vanished. The problem is objectified and ascribed to an internal malfunctioning, with the result that the client now appears as the sole responsible carrier of an illness. A challenge—for clinicians and researchers alike—is to understand “personality disorder” as not a fixed internal trait, but as patterns of contextual interaction triggered by specifiable interactional conditions.

### Implications for Practice

The couple therapy meetings with patients who have narcissistic problems pose specific challenges for the therapist. Addressing the client’s personal affairs, problems, and vulnerability may be perceived as threatening and may lead directly to resistance and to obstructive, non-cooperative responses. But addressing the client’s practices of interactional control and marking of independence may trigger balking and competitive reactions. A lesson that can be taken from our study is that in our view it is advisable for therapists, working with a client who shows narcissistic problems, to organize the first couple therapy session in an unobtrusive mode and to exercise their institutional authority in a weakened manner for the moment being. This “open” interactional strategy may, of course, also be perceived as threatening (as we have shown in section “Controlling the Sequential Position”), but it increases the chances that the client by him-/herself will find ways of cautious participation that can in the further process develop into more unrestricted and non-controlling co-operation.

Another challenge in couple therapy with clients having narcissistic problems lies in therapists experiencing confusion, having the feeling that it is difficult to work with these clients. The therapists may not be able to pinpoint the practices generating this impression. The results of our study should enable therapists to disentangle their intuitive understanding, i.e., to discern and identify in the course of interaction itself, the activities and phenomena that cause their uneasiness. Knowledge about the controlling practices of clients with narcissistic problems will help therapists to work with their own internal and interactional responses. Thereby, they may become able to better regulate their own input to the problematic interactions.

## Data Availability Statement

The raw data supporting the conclusions of this article will be made available by the authors, without undue reservation.

## Ethics Statement

The studies involving human participants were reviewed and approved by Bioethics Committee of Collegium Medicum at Jagiellonian University. The patients/participants provided their written informed consent to participate in this study.

## Author Contributions

All authors listed have made a substantial, direct and intellectual contribution to the work, and approved it for publication.

## Conflict of Interest

The authors declare that the research was conducted in the absence of any commercial or financial relationships that could be construed as a potential conflict of interest.

## Footnotes

1 Many of the first generation conversation analysts (Sacks, Schegloff, Sudnow, Turner) originally were students of Goffman, but in their groundbreaking work they were strongly influenced by Garfinkel’s (1967) program of ethnomethodology.

2 This ethnomethodological shift in asking is inspired by Moerman, who has done field research with the Lue tribe in Thailand and who showed that the question “who are the Lue?” needs to be reformulated and substituted by the question “When are the Lue?” (Moerman, 1974, p. 66).

3 Originally the data set also included two crisis couples. In the course of analysis these two couples were excluded and only used as a heuristic medium for comparison.

4 The project got agreement of the Bioethical Committee, Medical College, Jagiellonian University nr 1072.6120.76.2020.

5 An even stronger characterization of questions as a tool of power can be found in Canetti’s (1982) “Crowd and Power”: *”All questioning is a forcible intrusion. When used as an instrument of power it is like a knife cutting into the flesh of the victim”* (p. 284).

6 On jokes and funny remarks as “exit devices” in interaction, see Haugh and Musgrave (2018).

7 The distinction between “told” and “performed” self is linked to a question about the place of “content” of talk in CA. Although CA follows Wittgenstein’s dictum that “the meaning of a word is its use in language” (Wittgenstein, 2002 [1953], p. 18), it also pursues the question, how a content is formulated, e.g., in the use of “membership categories” or in practices of “formulating” such as “formulating place” (Schegloff, 1972) or “formulating planes” (Goodwin and Goodwin, 1996).

8 We want to stress that our notion of “anticipation” is not cognitivistic, but refers to the interactants’ expectations and perceptions of the future which is one of the “contexts” to which they are oriented in their actions; see the early paper of Drew (1995) on “anticipatory interactional planning” or Streeck and Jordan (2009) on the “forward-looking nature of embodied communication.”

9 A famous statement of Garfinkel(1963, p. 190), that became a cornerstone of ethnomethodology’s self-conception, is: “There is no reason to look under the skull since nothing of interest is to be found there but brains.”

10 With regard to the strangeness of others, Husserl(1960, p. 114) spoke of the “verifiable accessibility of what is not originally accessible.” Recent heated debates about “epistemics” between ethnomethodologists and conversation analysts show that there is much room for divergent understandings of such a perspective; see the discussions in the journal “Discourse Studies” 2013 and 2016.

## Appendix

**TABLE TA1:** Transcription symbols.
